# C-Reactive Protein-to-Albumin Ratio and Clinical Outcomes in COVID-19 Patients: A Systematic Review and Meta-Analysis

**DOI:** 10.3390/tropicalmed7080186

**Published:** 2022-08-16

**Authors:** Hernán J. Zavalaga-Zegarra, Juan J. Palomino-Gutierrez, Juan R. Ulloque-Badaracco, Melany D. Mosquera-Rojas, Enrique A. Hernandez-Bustamante, Esteban A. Alarcon-Braga, Vicente A. Benites-Zapata, Percy Herrera-Añazco, Adrian V. Hernandez

**Affiliations:** 1 Escuela de Medicina, Universidad Peruana de Ciencias Aplicadas, Lima 15023, Peru; 2 Sociedad Científica de Estudiantes de Medicina de la Universidad Peruana de Ciencias Aplicadas, Lima 15023, Peru; 3 Sociedad Científica de Estudiantes de Medicina de la Universidad Nacional de Trujillo, Trujillo 13011, Peru; 4 Grupo Peruano de Investigación Epidemiológica, Unidad para la Generación y Síntesis de Evidencias en Salud, Universidad San Ignacio de Loyola, Lima 15012, Peru; 5 Unidad de Investigación para la Generación y Síntesis de Evidencias en Salud, Vicerrectorado de Investigación, Universidad San Ignacio de Loyola, Lima 15012, Peru; 6 Escuela de Enfermería, Universidad Privada San Juan Bautista, Lima 15067, Peru; 7 Instituto de Evaluación de Tecnologías en Salud e Investigación—IETSI, EsSalud, Lima 14072, Peru; 8 Unidad de Revisiones Sistemáticas y Meta-Análisis, Guías de Práctica Clínica y Evaluaciones de Tecnología Sanitaria, Vicerrectorado de Investigación, Universidad San Ignacio de Loyola, Lima 15012, Peru; 9 Health Outcomes, Policy, and Evidence Synthesis (HOPES) Group, School of Pharmacy, University of Connecticut, Storrs, CT 06269, USA

**Keywords:** COVID-19, C-reactive protein, albumin, meta-analysis

## Abstract

C-reactive protein-to-albumin ratio (CAR) is an independent risk factor in cardiovascular, cerebrovascular, and infectious diseases. Through this study, we investigated the CAR values with respect to the severity and mortality of COVID-19 patients. We performed a systematic review and meta-analysis to retrieve studies that evaluated CAR values upon hospital admission in relation to the severity or mortality of COVID-19 patients. We adopted a random-effect model to calculate the pooled mean difference (MD) and their 95% confidence intervals (CI). Quality assessment was appraised using a Newcastle–Ottawa scale and publication bias was assessed using the Begg-test and funnel plot. We equally performed a subgroup analysis using study location and a sensitivity analysis only with studies with low risk of bias. We analyzed 32 studies (n = 12445). Severe COVID-19 patients had higher on-admission CAR values than non-severe COVID-19 patients (MD: 1.69; 95% CI: 1.35–2.03; *p* < 0.001; I^2^ = 89%). Non-survivor patients with COVID-19 had higher CAR values than survivor patients (MD: 2.59; 95% CI: 1.95–3.23; *p* < 0.001; I^2^ = 92%). In sensitivity analysis, the relationship remained with a decreasing of heterogeneity for severity (MD: 1.22; 95% CI: 1.03–1.40; *p* < 0.001; I^2^ = 13%) and for mortality (MD: 2.99; 95% CI: 2.47–3.51; *p* < 0.001; I^2^ = 0%). High CAR values were found in COVID-19 patients who developed severe disease or died.

## 1. Introduction

In December 2019, a new type of Coronavirus was identified that caused a disease similar to that caused by Severe Acute Respiratory Syndrome Coronavirus (SARS-CoV) in the 2002–2003 epidemic. This new virus was ultimately called Severe Acute Respiratory Syndrome Coronavirus 2 (SARS-CoV-2) and the disease it causes was called Coronavirus disease 2019 (COVID-19). On 11 March 2020, the WHO declared this disease a pandemic, and many measures were taken to prevent its transmission [1]. To this day (11 December 2021), more than 216 million cases and 4.4 million deaths have been reported worldwide [2]. However, despite many advances in the treatment and prevention of the disease, it is very difficult to accurately predict the severity of the symptoms that may lead to death [3].

COVID-19 causes an abnormal response of the host’s immune system. This causes the patient to develop many symptoms that eventually progress to severe pneumonia, multiple organ failure, septic shock, and death. Risk factors that increase the likelihood of death are as follows: being a male, age over 65 years, smoking, and possessing comorbidities such as hypertension, diabetes, and cardiovascular and respiratory diseases [4,5].

During the pandemic, some clinicians could not accurately predict if a specific patient could develop a more serious form of the disease or if there was a high chance that the patient will die. However, many studies and meta-analyses have been performed that have proposed different biomarkers for poor prognosis for COVID-19, including C-reactive protein (CRP), albumin, procalcitonin, neutrophil-to-lymphocyte ratio, apolipoproteins, D-dimer, and ferritin [6,7,8,9].

C-reactive protein-to-albumin ratio (CAR) is an accessible biomarker because CRP and albumin are widely used in most healthcare centers. To date, there is no consensus on the normal values of CAR. However, some studies showed the benefits of CAR as an inflammation-related indicator of prognosis for cardiovascular, cerebrovascular, and infectious diseases [10,11,12]. This is because CRP increases its values in the acute inflammatory response to viruses, whereas the production of albumin, another kind of protein, is decreased under the same conditions [13]. In order to give clinicians and other healthcare personnel a good and reliable tool to predict the severity of COVID-19, we investigated the values of CAR in relation to severity or mortality in such patients.

## 2. Methods

### 2.1. Study Design, Register, and Report

This systematic review was performed according to Cochrane Collaboration guidance and the Preferred Reporting Items for Systematic Reviews and Meta-Analyses (PRISMA) statement (See PRISMA checklist in Supplementary Table S1) [14]. A short version of the protocol was submitted in the International Prospective Register of Systematic Reviews (PROSPERO) with identification code: CRD42021279087.

### 2.2. Search Strategy and Data Sources

The literature search was focused on retrieving articles reporting CAR values in COVID-19 patients (See File S1: Search Strategy in Supplementary Material). Considering the Peer Review of Electronic Search Strategies checklist [15], the search strategy was built for PubMed using MeSH and free terms, and was adapted for the following databases: Scopus, Web of science, Embase, LILACS, The Cochrane Library, and the WHO COVID-19 Global Research Database. Moreover, a hand-search was performed in preprint platforms (MedRxiv, Authorea, and Research Square) and other sources (CDC COVID-19 Research Article Database and CNKI databases). This took place on 7 June 2022 and no language restriction was applied for this systematic review.

### 2.3. Eligibility Criteria

This systematic review included studies describing the relationship between CAR values and COVID-19 severity or mortality, with case–control or cohort designs, and that were conducted in patients over 18 years of age who were diagnosed with COVID-19. We excluded duplicates, studies with participants that did not meet all eligibility criteria, and studies with wrong exposures.

### 2.4. Study Selection

Four reviewers (HJZ-Z, JJP-G, EAH-B, and JRU-B) independently screened all records from the systematic search using titles and abstracts. Afterward, the remaining records were fully reviewed and studies with participants meeting all eligibility criteria were included. Any conflict in the process of study selection was resolved by consensus among the authors. Software Rayyan QCRI (Rayyan Systems Inc ©, Cambridge, MA, USA) was used for deleting duplicates and screening titles and abstracts [16].

### 2.5. Data Extraction

Four authors collected data from the included studies in a Microsoft Excel © 2013 data extraction sheet (HJZ-Z, JJP-G, MDM-R, EAA-B). These include study titles, first author, publication date, study location, study design, baseline characteristics (sample size, age, sex, and any subgroup) of the study population, exposure measurements (CAR means or medians values, optimal CAR cutoff values, area under the curve (AUC), sensitivity, and specificity), and outcomes (severity or mortality).

### 2.6. Quality Assessment

Four authors performed quality assessment (HJZ-Z, JJP-G, EAA-B, and EAH-B) of the data collected. The Newcastle–Ottawa scale (NOS) was used to determine the risk of bias from all included studies and these studies were categorized as follows: low risk (≥6), moderate risk (4–5) and high risk (≤3) [17].

### 2.7. Data Synthesis and Statistical Analysis

We converted variables presented as medians and interquartile ranges (IQR) to means and standard deviation (SD), respectively, using Wan’s method [18]. We equally used the mean difference (MD) and SD from each study to estimate the pooled MD with 95% confidence intervals (CI). Review Manager 5.4 (RevMan 5.4) (The Cochrane Collaboration, Copenhagen, Denmark) was used for statistical analysis. Statistical heterogeneity was determined using I^2^ statistics and a Cochran Q-test. Heterogeneity was categorized as severe (I^2^ ≥ 60%) or not severe (I^2^ < 60%). A random-effect model, a subgroup analysis according to study location, and a sensitivity analysis using only studies with low risk of bias were performed due to anticipated heterogeneity. A *p*-value < 0.1 was considered statistically significant. The primary outcome was severe disease which was defined as meeting at least one of the following criteria: respiration rate ≥ 30 cycles per minute, ICU admission, blood oxygen saturation at rest ≤ 93%, shortness of breath, and PaO2/FiO2 ≤ 300 mm Hg. Mortality was considered a secondary outcome. However, the definitions proposed by the articles were also considered.

### 2.8. Publication Bias

Publication bias was assessed using Begg’s test and illustrated in funnel plots. Moreover, *p*-values > 0.1 signified no publication bias.

## 3. Results

### 3.1. Study Selection

**Figure 1:**
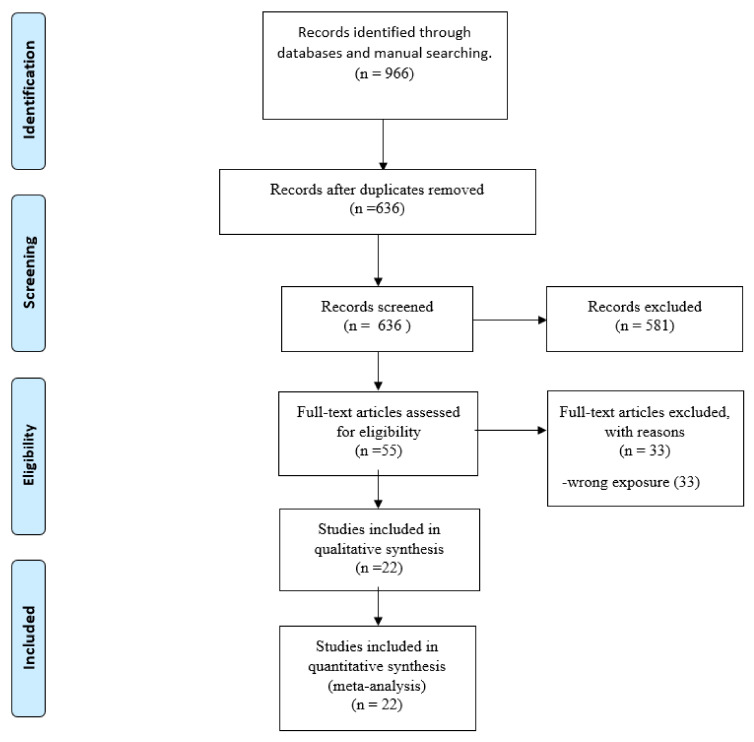
PRISMA Flow Diagram.

Our systematic search retrieved 966 articles. After excluding duplicates and screening titles and abstracts, 55 articles remained for full-text review. Furthermore, 32 articles were maintained after full-text screening with all eligibility criteria [19,20,21,22,23,24,25,26,27,28,29,30,31,32,33,34,35,36,37,38,39,40,41,42,43,44,45,46,47,48,49,50]. The process of study selection was summed up in a flow diagram (Figure 1).

### 3.2. Study Characteristics

All 32 included studies were cohorts; 15 reported on the severity of COVID-19 patients only, 13 reported on the mortality of COVID-19 patients, and 4 reported on both outcomes (severity and mortality). Arslan K et al. [43] authored the largest cohort with 1579 participants and Paliogiannis P et al. [31] authored the smallest cohort with 30 participants. All included studies were conducted and published between 2020 and 2022. According to study location, 21 studies were carried out in Turkey, 6 in China, 2 in Egypt, 1 in India, 1 in the USA, and 1 in Italy.

There were 14 studies that evaluated optimal CAR cutoff values and AUC for severity, ranging from 0.296 to 4.2 and 0.107to 0.934, respectively. Meanwhile, the optimal CAR cutoff values and AUC for mortality was assessed in 11 studies, ranging from 0.34 to 4.21 and 0.767 to 0.862, respectively. All included studies summed up to a population of 12445 COVID-19 patients; 6924 were male patients whose age ranged from 19 to 99 years. We summarized these characteristics in Table 1 and Table 2.

### 3.3. Quality Assessment

After assessing the risk of bias using the NOS (see Supplementary Table S2), 12 studies were at low risk, 11 moderate risk and 9 were at high risk. 

### 3.4. CAR and COVID-19 Severity

**Figure 2:**
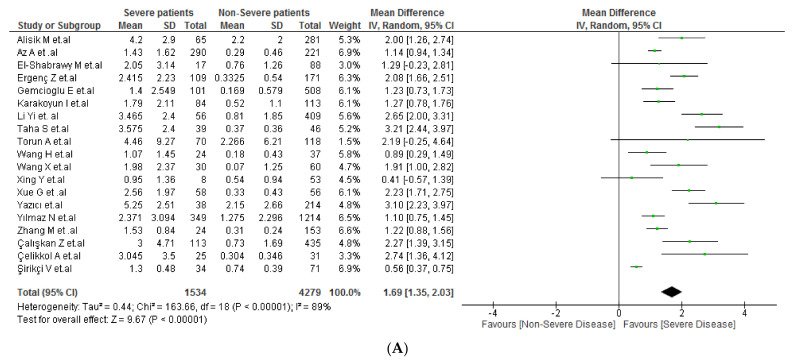

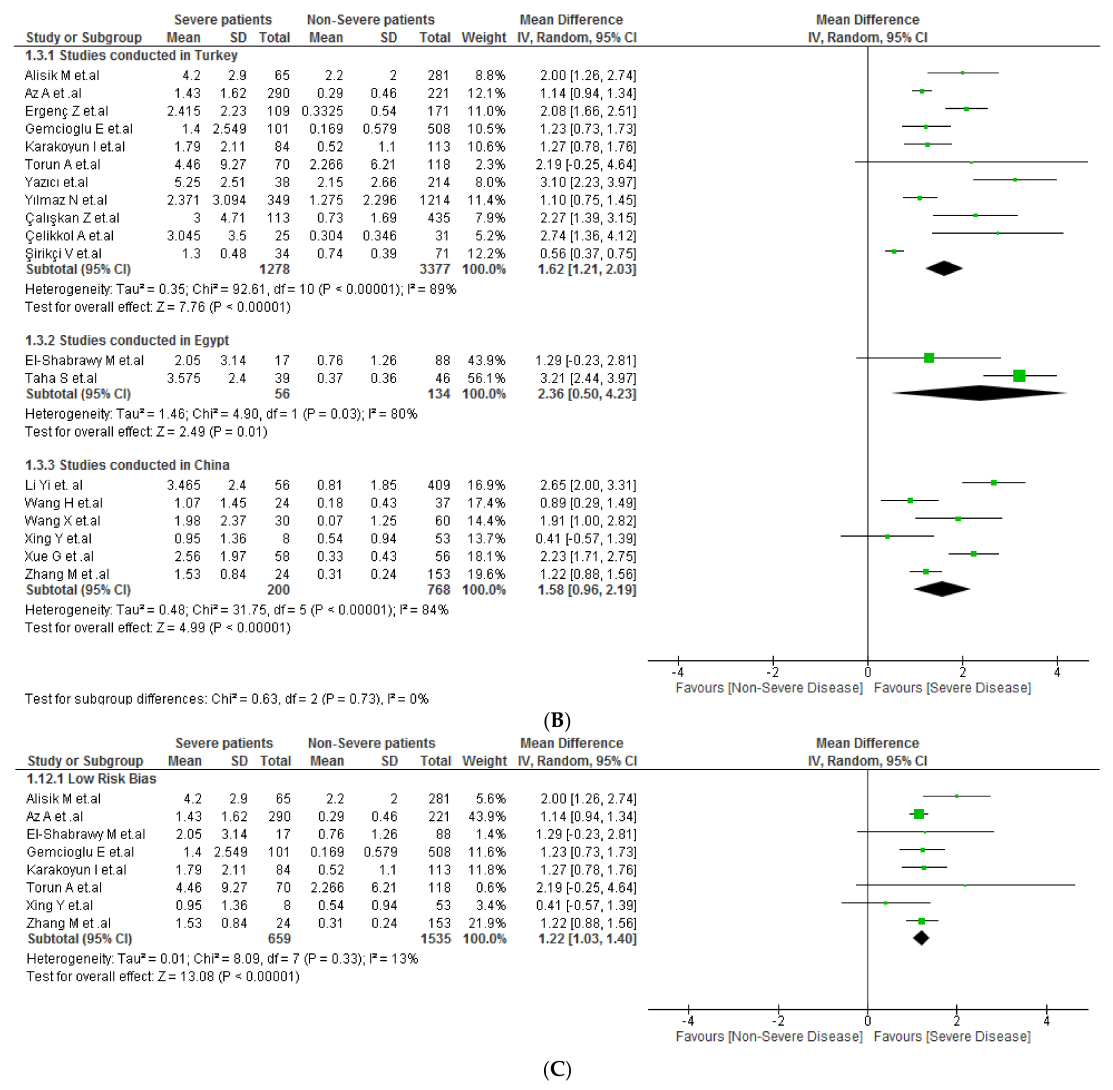
(**A**) CAR values in severe vs. non-severe COVID-19 patients. [19,22,23,24,25,28,33,34,35,36,37,38,39,40,41,47,48,49,50] (**B**) Subgroup analysis according to country of origin between severe vs. non-severe COVID-19 patients. [19,22,23,24,25,28,33,34,35,36,37,38,39,40,41,47,48,49,50]. (**C**) Sensitivity analysis according to the risk of bias between severe vs. non-severe COVID-19 patients [19,22,23,24,25,28,35,38].

The relationship between COVID-19 severity and CAR was analyzed in 19 studies with a population of 5813 patients (2141 patients developed severe disease). Severe patients had higher CAR values than non-severe patients (MD: 1.69; 95% CI: 1.35–2.03; *p* < 0.001) (Figure 2A). In addition, a subgroup analysis was performed by study location due to severe heterogeneity (I^2^ = 89%). Significant differences and severe heterogeneities were found in Turkish studies (MD: 1.62; 95% CI: 1.21–2.03; *p* < 0.001; I^2^ = 89), Egyptian studies (MD: 2.36; 95% CI: 0.5–4.23; *p* < 0.001; I^2^ = 80) and Chinese studies (MD: 1.58; 95% CI: 0.96–2.19; *p* < 0.001; I^2^ = 84) with an interaction test, *p* = 0.26 (Figure 2B). Sensitivity analysis was performed with studies having a low risk of bias and the relationship observed previously between CAR values and COVID-19 severity was similar (MD: 1.22; 95% CI: 1.03–1.40; *p* < 0.001). However, heterogeneity decreased significantly with sensitivity analysis (I^2^ = 13%) (Figure 2C).

### 3.5. CAR and COVID-19 Mortality

**Figure 3:**
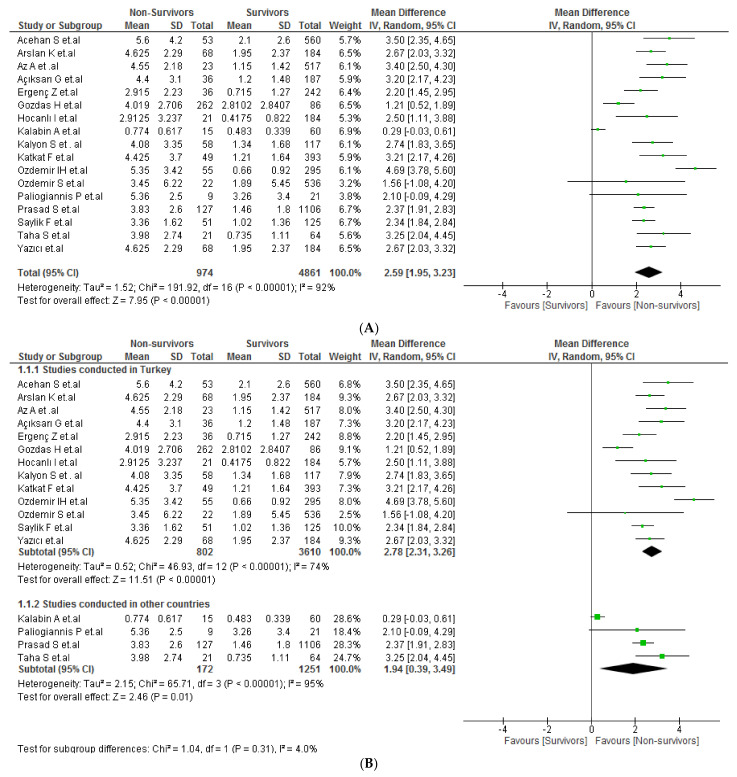

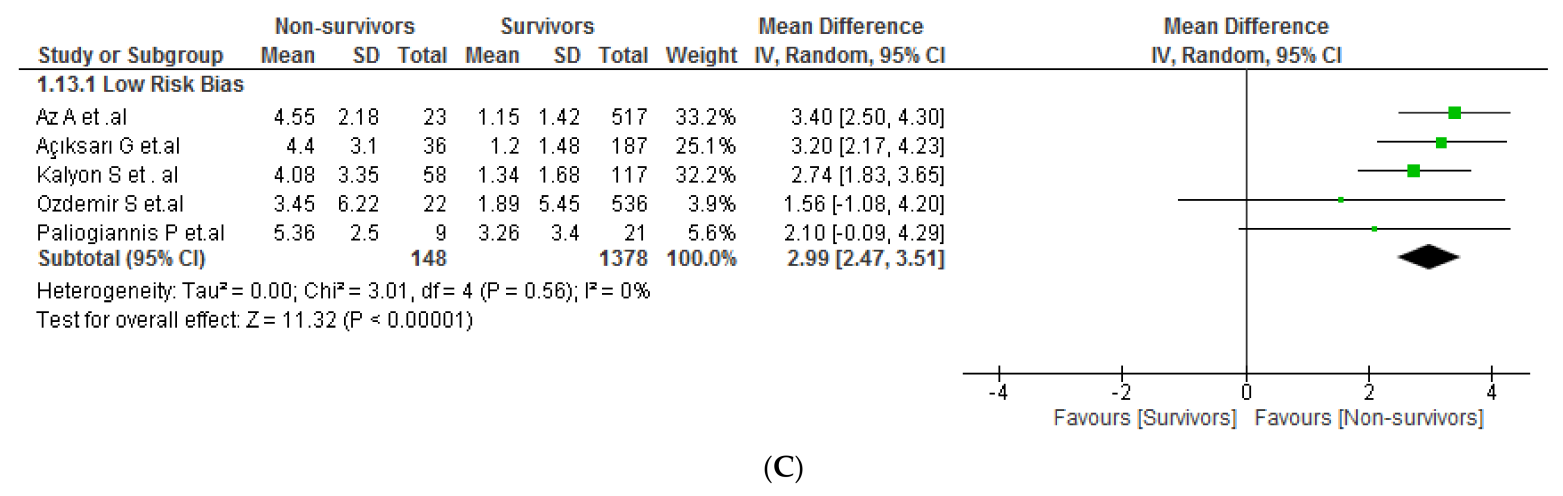
(**A**) CAR values in non-survivor vs. survivor COVID-19 patients [20,21,23,26,27,29,30,31,32,34,43,44,45,46,47,50]. (**B**) Subgroup analysis according to country of origin between non-survivor vs. survivor COVID-19 patients. [20,21,23,26,27,29,30,31,32,34,43,44,45,46,47,50] (**C**) Sensitivity analysis according to the risk of bias between non-survivor vs. survivor COVID-19 patients [21,23,27,30,31].

COVID-19 mortality and CAR values were assessed in 17 studies with a population of 7164 patients. Non-survivor patients had higher CAR values than survivor patients (MD: 2.59; 95% CI: 1.95–3.23; *p* < 0.001) (Figure 3A). Due to severe heterogeneity (I^2^ = 92%), we performed a subgroup analysis based on study location (Figure 3B). Significant differences were found in the Turkish studies only (MD: 2.78; 95% CI: 2.31–3.26; *p* < 0.001) with severe heterogeneity (I^2^ = 74%). Likewise, sensitivity analysis included only studies with low risk of bias and demonstrated the same relationship (MD: 2.99; 95% CI: 2.47–3.51; *p* < 0.001). In addition, heterogeneity decreased significantly in this way (I^2^ = 0%) (Figure 3C).

### 3.6. Publication Bias

Publication bias was performed using the Begg’s tests for COVID-19 severity and mortality. As a result, no indication of small study effects (*p* = 0.584 and *p* = 0.474, respectively) were observed. In addition, funnel plot for the included studies showed a symmetric pattern (see Supplementary Figures S1 and S2)

## 4. Discussion

From our study, we noted that patients with severe COVID-19 (alongside those who died due to COVID-19) had higher CAR values at admission than those who survived or did not develop severe disease.

In patients with COVID-19, the activation of inflammatory signals and the cytokine storm are crucial in the development of acute respiratory distress syndrome [51]. In these patients, the massive production of cytokines and chemokines causes a dysregulation of the innate immune system and attracts inflammatory cells that infiltrate lung tissue as they cause immunological damage [51]. Thus, inflammation is a marker of severity and prognosis in these patients. A systematic review and metanalysis of 23 studies demonstrated that patients with severe disease had higher values of procalcitonin, CRP, D-dimer and LDH, and lower levels of albumin compared to that observed in non-severe patients [52]. Moreover, another meta-analysis of 17 articles revealed a marked decrease in lymphocytes, monocytes, eosinophils, platelets, albumin, CRP-to-lymphocyte ratio, and CRP-to-leukocyte ratio. In addition, it projected high values of PCR, ESR, procalcitonin, LDH, and others [53]. The effect of inflammation in patients with COVID-19 necessitates the search for more stable markers that can accurately predict the prognosis, severity, and mortality of affected patients [54]. This prompted our study and, from the findings above, we observed that there is an association between CAR and severity/mortality of COVID-19 patients.

CAR is a known marker in different clinical scenarios associated with inflammation. Moreover, many systematic reviews showed the prognostic value of CAR in different types of cancer because of the strong relationship between inflammation and carcinogenesis [55,56,57,58]. In the same way, inflammation usually results in the loss of muscular mass in malnourished patients undergoing hemodialysis [59]. Inflammation is not exclusive to viral infections and could increase in severe situations such as in sepsis [60] or critically ill patients [61,62]. This also explains our findings, as severity and mortality in COVID-19 patients are usually associated with sepsis or critical illness [63,64]. Likewise, similar findings were observed in a study evaluating the association between CAR and the prognosis of patients with pneumonia of an etiology other than COVID-19 [65], thus explaining our results in patients with COVID-19 pneumonia [63,64].

In cerebrovascular and cardiovascular diseases, inflammation is involved in their pathogenesis, thereby explaining the association between CAR and mortality in patients with acute coronary syndrome [66], brain ischemia [67], peripheric arterial disease [68], abdominal aortic aneurism [69] or atrial fibrillation after a coronary bypass [70]. This also explains our findings considering the brain and cardiovascular complications that occur in patients with COVID-19. These included myocarditis, acute myocardial infarction, heart failure, arrythmia, and thromboembolic events [71,72], episodes of stroke, and necrotizing hemorrhagic encephalitis, among others [73,74]. Our study is the first systematic review that evaluates CAR values in the mortality and severity of COVID-19. Moreover, we used the NOS to evaluate bias risk in the included articles and we performed a sensibility analysis considering these bias, thereby rendering our findings more reliable.

To our knowledge, there are no studies that have compared CAR with other similar markers in COVID-19 patients. However, some studies in other pathologies demonstrate that CAR has a better predictive value than other markers [75,76]. Therefore, our findings elucidate that CAR is a low-cost prognostic marker in patients admitted for COVID-19 and provides a clue for health personnel to prioritize or individualize management strategies in patients with high CAR values. Our systematic review and metanalysis revealed a variability for CAR cutoffs in included studies. Thus, further research is needed to define optimal CAR cutoffs for different populations to stratify risk for severity or mortality in COVID-19 patients. However, it is possible that the prognostic value of the CAR varies according to the type of patient, for example, in patients with low albumin values, such as patients with cirrhosis [77] or nephrotic syndrome [78]. Nevertheless, although there are no studies in patients with proteinuria, many studies demonstrate that CAR is a good predictor of mortality in patients with decompensated cirrhosis, a condition that involves low albumin values [79]. This reveals that even in patients with COVID-19, it is possible to use the CAR as a prognostic marker.

### Limitations

Our findings must be interpreted within the context of their own limitations. Firstly, the high statistical heterogeneity obtained after meta-analysis resulted from clinical and methodological differences from included studies. Nevertheless, heterogeneity could also be explained by differences between study locations and risk of bias. Secondly, estimated effect measures were calculated as mean differences without adjusting for potential confounders such as age, sex, or comorbidities, which may influence inflammatory processes such as sepsis. This lack of adjustment may explain the heterogeneity of the results. However, elevated CAR values at hospital admission were consistently associated with COVID-19 severity or mortality. Thirdly, studies that calculated a CAR cutoff did not report the incidence of severe disease or mortality in relation to these cutoff points. If incidence had been reported, the relative risks (a measure of association easier to interpret by clinicians than mean differences) could easily be deduced. Fourth, our systematic search had no language restrictions; however, most included studies were conducted in Asia. Our study provides relevant information about the general potential role of CAR as a marker for COVID-19 severity or mortality. However, physiological or geographical variations of CRP and albumin levels could cause differences in assessed outcomes. In this sense, further research is needed to evaluate the clinical role of CAR, adjusting for these variables in COVID-19 patients. Fifth, we cannot ascertain that this marker is better than others in assessing the prognosis of patients with COVID-19. This issue should be approached using predictive models that assess several inflammation markers for COVID-19.

## 5. Conclusions

High CAR values were found in admitted patients who died or developed severe disease. However, further research is needed to establish an optimal cutoff value of CAR that can accurately predict severity and mortality in COVID-19 patients.

## Figures and Tables

**Table 1:** Characteristics of included studies comparing severe and non-severe COVID-19 patients.

Author	Year	Country	Participants (Male)	Mean/Median Age (IQR/SD)	Outcome	CAR Mean (SD) in Severe Patients	CAR Mean (SD) in Non-Severe Patients	CAR Cutoff Value	Area Under the Curve (AUC)	Sensitivity (%)	Specificity (%)
**Zhang M et al. [19]**	2020	China	177 (99)	42 (15)	Severity	1.53 (0.84)	0.31 (0.24)	0.73	0.908	79.2	95.1
**Karakoyun I et al. [28]**	2021	Turkey	197 (108)	54 (18)	Severity	1.79 (2.11)	0.52 (1.1)	0.9	0.718	69.1	70.8
**El-Shabrawy M et al. [24]**	2021	Egypt	116 (63)	54 (20–88)	Severity	2.05 (3.14)	0.76 (1.26)	0.89	0.922	82.4	90.9
**Wang X et al. [36]**	2020	China	90 (48)	63 (46–84)	Severity	1.98 (2.37)	0.07 (0.125)	0.296	0.812	76.7	80.4
**Xue G et al. [39]**	2020	China	114 (64)	62 (51–70)	Severity	2.56 (1.97)	0.33 (0.43)	0.71	0.81	82.76	80.36
**Wang H et al. [37]**	2020	China	61 (31)	53 (40–62)	Severity	1.07 (1.45)	0.18 (0.43)	NR	NR	NR	NR
**Torun A et al. [35]**	2021	Turkey	188 (93)	60 (12)	Severity	4.46 (9.27)	2.266 (6.21)	0.754	0.841	82.6	66.7
**Gemcioglu E et al. [25]**	2021	Turkey	609 (348)	49 (26.5)	Severity	1.4 (2.549)	0.169 (0.579)	0.625	0.765	68.32	75.49
**Yılmaz N et al. [40]**	2021	Turkey	1563 (925)	51 (19.5)	Severity	2.37 (3.09)	1.27 (2.29)	NR	NR	NR	NR
**Şirikçi V et al. [33]**	2021	Turkey	105 (39)	63 (14)	Severity	1.3 (0.48)	0.74 (0.39)	1	0.7	76.5	76.1
**Xing Y et al. [38]**	2020	China	61 (31)	53 (41–63)	Severity	0.95 (1.36)	0.54 (0.94)	NR	NR	NR	NR
**Alisik M et al. [22]**	2021	Turkey	326 (168)	51 (35–68)	Severity	4.2 (2.9)	2.2 (2)	1.21	0.86	86.2	75.9
**Li Y et al. [41]**	2021	China	465 (248)	62 (54–69)	Severity	3.465 (2.4)	0.81 (1.85)	1.843	0.107	NR	NR
**Taha S et al. [34]**	2021	Egypt	85 (48)	55 (42–65).	Severity Mortality	3.575 (2.4)	0.37 (0.36)	1.65	0.878	76.9	95.7
**Az A et al. [23]**	2021	Turkey	540 (302)	48 (14.6)	Severity Mortality	1.43 (1.62)	0.29 (0.46)	NR	NR	NR	NR
**Çelikkol A et al. [49]**	2022	Turkey	56 (23)	47.5 (18.8)	Severity	3.045 (3.4)	0.304(0.346)	0.475	0.934	90.91	86.21
**Çalışkan Z et al. [48]**	2022	Turkey	548 (286)	64 (21)	Severity	3 (4.71)	0.73 (1.69)	2.19	0.763	78.55	63.11
**Ergenç Z et al. [47]**	2022	Turkey	280 (133)	58.34 (18.64)	Severity Mortality	2.415 (2.23)	0.3325 (0.54)	NR	NR	NR	NR
**Yazıcı et al. [50]**	2022	Turkey	252 (107)	77 (70–83)	Severity Mortality	5.25 (2.51)	2.15 (2.66)	4.2	0.786	73.7	75.2

**Table 2:** Characteristics of the included studies that evaluated the mortality.

Author	Year	Country	Participants (Male)	Mean/Median Age (IQR/SD)	Outcome	CAR Mean (SD) in Non-Survivors Patients	CAR Mean (SD) in Survivors Patients	CAR Cutoff Value	Area Under the Curve (AUC)	Sensitivity (%)	Specificity (%)
**Taha S et al. [34]**	2021	Egypt	85 (48)	55 (42–65).	Mortality Severity	3.98 (2.74)	0.735 (1.11)	4.21	0.812	57.1	90.6
**Az A et al. [23]**	2021	Turkey	540 (302)	48 (14.6)	Mortality Severity	4.55 (2.18)	1.15 (1.42)	NR	NR	NR	NR
**Açıksarı G et al. [21]**	2021	Turkey	223 (118)	60 (19)	Mortality	4.4 (3.1)	1.2 (1.48)	0.34	0.81	NR	NR
**Saylik F et al. [32]**	2021	Turkey	176 (51)	64 (10)	Mortality	3.36 (1.62)	1.02 (1.36)	2.075	0.778	82.3	72.8
**Kalabin A et al. [26]**	2021	United States of America	75 (49)	63 (14)	Mortality	0.774 (0.617)	0.483 (0.339)	0.54	NR	NR	NR
**Paliogiannis P et al. [31]**	2020	Italy	30 (16)	72 (65–68)	Mortality	5.36 (2.5)	3.26 (3.4)	NR	NR	NR	NR
**Kalyon S et al. [27]**	2020	Turkey	175 (72)	73 (65–95)	Mortality	4.08 (3.35)	1.34 (1.68)	2.3	0.781	70.69	72.65
**Özdemir IH et al. [29]**	2021	Turkey	350 (194)	55 (39–70)	Mortality	5.35 (3.42)	0.66 (0.92)	NR	NR	NR	NR
**Özdemir S et al. [30]**	2021	Turkey	558 (310)	48 (19–96)	Mortality	3.45 (6.22)	1.89 (5.45)	NR	NR	NR	NR
**Acehan S et al. [20]**	2021	Turkey	613 (358)	59 (19.5)	Mortality	5.6 (4.2)	2.1 (2.6)	2.1561	0.79	73.6	68.4
**Katkat F et al. [46]**	2022	Turkey	442 (247)	58 (18–99)	Mortality	4.425(3.7)	1.21(1.64)	2.2	0.809	76	75
**Prasad S et al. [45]**	2022	India	1233 (853)	53.5(15.79)	Mortality	3.83 (2.6)	1.46 (1.8)	2.08	0.794	70.1	27.2
**Hocanlı I et al. [44]**	2022	Turkey	205 (113)	53.5 (34.7–87)	Mortality	2.912 (3.23)	0.41 (0.82)	1.39	0.862	76	81
**Arslan K et al. [43]**	2022	Turkey	1579 (824)	54 (43–65)	Mortality	2.34 (1.08)	0.472 (0.8)	1.09	0.851	94.6	74.1
**Gozdas H et al. [42]**	2022	Turkey	348 (205)	74 (65–83)	Mortality	4.01 (2.70)	2.81 (2.84)	NR	NR	NR	NR
**Ergenç Z et al. [47]**	2022	Turkey	280 (133)	58.34 (18.64)	Mortality Severity	0.715 (1.27)	2.915 (2.23)	NR	NR	NR	NR
**Yazıcı et al. [50]**	2022	Turkey	252 (107)	77 (70–83)	Mortality Severity	4.625 (2.29)	1.95 (2.37)	3	0.767	76.5	70.1

## Data Availability

Not applicable.

## Supplementary Materials

The following supporting information can be downloaded at: https://www.mdpi.com/article/10.3390/tropicalmed7080186/s1, File S1: Search Strategy; Figure S1: Funnel Plot of the studies that evaluated CAR values in severe vs. non-severe COVID-19 patients; Figure S2: Funnel Plot of the studies that evaluated CAR values in non-survivor vs. survivor COVID-19 patients; Table S1: PRISMA Checklist; Table S2: Newcastle—Ottawa quality assessment scale for included studies

## References

[1] Bonilla-Aldana D.K., Quintero-Rada K., Montoya-Posada J.P., Ramírez-Ocampo S., Paniz-Mondolfi A., Rabaan A.A., Sah R., Rodríguez-Morales A.J. (2020). SARS-CoV, MERS-CoV and Now the 2019-Novel CoV: Have We Investigated Enough about Coronaviruses?—A Bibliometric Analysis. Travel Med. Infect. Dis..

[2] WHO Coronavirus (COVID-19) Dashboard | WHO Coronavirus (COVID-19) Dashboard with Vaccination Data. https://covid19.who.int/.

[3] Ray D., Salvatore M., Bhattacharyya R., Wang L., Du J., Mohammed S., Purkayastha S., Halder A., Rix A., Barker D. (2020). Predictions, Role of Interventions and Effects of a Historic National Lockdown in India’s Response to the COVID-19 Pandemic: Data Science Call to Arms. Harv. Data Sci. Rev..

[4] Li L.Q., Huang T., Wang Y.Q., Wang Z.P., Liang Y., Huang T.B., Zhang H.Y., Sun W., Wang Y. (2020). COVID-19 Patients’ Clinical Characteristics, Discharge Rate, and Fatality Rate of Meta-Analysis. J. Med. Virol..

[5] Tian W., Jiang W., Yao J., Nicholson C.J., Li R.H., Sigurslid H.H., Wooster L., Rotter J.I., Guo X., Malhotra R. (2020). Predictors of Mortality in Hospitalized COVID-19 Patients: A Systematic Review and Meta-analysis. J. Med. Virol..

[6] Ulloque-Badaracco J.R., Alarcon-Braga E.A., Hernandez-Bustamante E.A., Al-kassab-Córdova A., Mosquera-Rojas M.D., Ulloque-Badaracco R.R., Huayta-Cortez M.A., Maita-Arauco S.H., Herrera-Añazco P., Benites-Zapata V.A. (2022). Fibrinogen-to-Albumin Ratio and Blood Urea Nitrogen-to-Albumin Ratio in COVID-19 Patients: A Systematic Review and Meta-Analysis. Trop. Med. Infect. Dis..

[7] Ulloque-Badaracco J.R., Hernandez-Bustamante E.A., Herrera-Añazco P., Benites-Zapata V.A. (2021). Prognostic Value of Apolipoproteins in COVID-19 Patients: A Systematic Review and Meta-Analysis. Travel Med. Infect. Dis..

[8] Ulloque-Badaracco J.R., Salas-Tello W.I., Al-kassab-Córdova A., Alarcón-Braga E.A., Benites-Zapata V.A., Maguiña J.L., Hernandez A.V. (2021). Prognostic Value of Neutrophil-to-Lymphocyte Ratio in COVID-19 Patients: A Systematic Review and Meta-Analysis. Int. J. Clin. Pract..

[9] Ulloque-Badaracco J.R., Mosquera-Rojas M.D., Hernandez-Bustamante E.A., Alarcón-Braga E.A., Herrera-Añazco P., Benites-Zapata V.A. (2022). Prognostic Value of Albumin-to-Globulin Ratio in COVID-19 Patients: A Systematic Review and Meta-Analysis. Heliyon.

[10] Kim M.H., Ahn J.Y., Song J.E., Choi H., Ann H.W., Kim J.K., Kim J.H., Jeon Y.D., Kim S.B., Jeong S.J. (2015). The C-Reactive Protein/Albumin Ratio as an Independent Predictor of Mortality in Patients with Severe Sepsis or Septic Shock Treated with Early Goal-Directed Therapy. PLoS ONE.

[11] Luo B., Sun M., Huo X., Wang Y. (2021). Two New Inflammatory Markers Related to the CURB-65 Score for Disease Severity in Patients with Community-Acquired Pneumonia: The Hypersensitive C-Reactive Protein to Albumin Ratio and Fibrinogen to Albumin Ratio. Open Life Sci..

[12] Zhang D., Yan H., Wei Y., Liu X., Zhuang Z., Dai W., Li J., Li W., Hang C. (2019). C-Reactive Protein/Albumin Ratio Correlates With Disease Severity and Predicts Outcome in Patients With Aneurysmal Subarachnoid Hemorrhage. Front. Neurol..

[13] Feketea G.M., Vlacha V. (2020). The Diagnostic Significance of Usual Biochemical Parameters in Coronavirus Disease 19 (COVID-19): Albumin to Globulin Ratio and CRP to Albumin Ratio. Front. Med..

[14] Liberati A., Altman D.G., Tetzlaff J., Mulrow C., Gøtzsche P.C., Ioannidis J.P.A., Clarke M., Devereaux P.J., Kleijnen J., Moher D. (2009). The PRISMA Statement for Reporting Systematic Reviews and Meta-Analyses of Studies That Evaluate Healthcare Interventions: Explanation and Elaboration. BMJ.

[15] McGowan J., Sampson M., Salzwedel D., Cogo E., Foerster V., Lefebvre C. (2016). PRESS Peer Review of Electronic Search Strategies: 2015 Guideline Statement. J. Clin. Epidemiol..

[16] Ouzzani M., Hammady H., Fedorowicz Z., Elmagarmid A. (2016). Rayyan—A Web and Mobile App Forsystematic Reviews. Syst. Rev..

[17] Wells G., Shea B., O’Connell D., Peterson J., Welch V., Losos M., Tugwell P. The Newcastle-Ottawa Scale (NOS) for Assessing the Quality of Nonrandomised Studies in Meta-Analyses. http://www.ohri.ca/programs/clinical_epidemiology/oxford.asp.

[18] Wan X., Wang W., Liu J., Tong T. (2014). Estimating the Sample Mean and Standard Deviation from the Sample Size, Median, Range and/or Interquartile Range. BMC Med. Res. Methodol..

[19] Zhang M., Xiao E., Liu J., Cai Y., Yu Q. (2020). An Emerging Marker Predicting the Severity of COVID-19: Neutrophil-Lymphocyte Count Ratio. ResearchSquare.

[20] Acehan S., Gülen M., Işıkber C., Kaya A., Unlu N., İnce Ç., Toptaş Fırat B., Köksaldı Şahin G., Erdem Sümbül H., Satar S. (2021). C-Reactive Protein to Albumin Ratio Is Associated with Increased Risk of Mortality in COVID-19 Pneumonia Patients. Cukurova Med. J..

[21] Açıksarı G., Koçak M., Çağ Y., Altunal L.N., Atıcı A., Çelik F.B., Bölen F., Açıksarı K., Çalışkan M. (2021). Prognostic Value of Inflammatory Biomarkers in Patients with Severe COVID-19: A Single-Center Retrospective Study. Biomark. Insights.

[22] Alisik M., Erdogan U., Ates M., Sert M., Yis O., Bugdayci G. (2021). Predictive Values of Immature Granulocyte and Other Inflammatory Parameters on Disease Severity of COVID-19 Patients. Int. J. Med. Biochem..

[23] Az A., Sogut O., Akdemir T., Ergenc H., Dogan Y., Cakirca M. (2021). Impacts of Demographic and Clinical Characteristics on Disease Severity and Mortality in Patients with Confirmed COVID-19. Int. J. Gen. Med..

[24] El-Shabrawy M., Alsadik M.E., El-Shafei M., Abdelmoaty A.A., Alazzouni A.S., Esawy M.M., Shabana M.A. (2021). Interleukin-6 and C-Reactive Protein/Albumin Ratio as Predictors of COVID-19 Severity and Mortality. Egypt. J. Bronchol..

[25] Gemcioglu E., Davutoglu M., Catalbas R., Karabuga B., Kaptan E., Aypak A., Kalem A.K., Ozdemir M., Yescedililova N.Y., Kalkan E.A. (2021). Predictive Values of Biochemical Markers as Early Indicators for Severe COVID-19 Cases in Admission. Future Virol..

[26] Kalabin A., Mani V.R.K., Valdivieso S.C., Donaldson B. (2021). Does C Reactive Protein/Albumin Ratio Have Prognostic Value in Patients with COVID-19. J. Infect. Dev. Ctries..

[27] Kalyon S., Gültop F., Şimşek F., Adaş M. (2021). Relationships of the Neutrophil-Lymphocyte and CRP-Albumin Ratios with the Duration of Hospitalization and Fatality in Geriatric Patients with COVID-19. J. Int. Med. Res..

[28] Karakoyun I., Colak A., Turken M., Altin Z., Arslan F.D., Iyilikci V., Yilmaz N., Kose S. (2021). Diagnostic Utility of C-Reactive Protein to Albumin Ratio as an Early Warning Sign in Hospitalized Severe COVID-19 Patients. Int. Immunopharmacol..

[29] Özdemir İ.H., Özlek B., Çetin N. (2021). Permanent Atrial Fibrillation Portends Poor Outcomes in Hospitalized Patients with COVID-19: A Retrospective Observational Study. J. Electrocardiol..

[30] Özdemir S., Akça H.Ş., Algın A., Altunok İ., Eroğlu S.E. (2021). Effectiveness of the Rapid Emergency Medicine Score and the Rapid Acute Physiology Score in Prognosticating Mortality in Patients Presenting to the Emergency Department with COVID-19 Symptoms. Am. J. Emerg. Med..

[31] Paliogiannis P., Zinellu A., Scano V., Mulas G., de Riu G., Pascale R.M., Arru L.B., Carru C., Pirina P., Mangoni A.A. (2020). Laboratory Test Alterations in Patients with COVID-19 and Non COVID-19 Interstitial Pneumonia: A Preliminary Report. J. Infect. Dev. Ctries.

[32] Saylik F., Akbulut T., Kaya S. (2021). Can C-Reactive Protein to Albumin Ratio Predict In-Hospital Death Rate Due to COVID-19 in Patients with Hypertension?. Angiology.

[33] Sirikci V., Findikli H.A., Erdogan M. (2021). The Relationship between Disease Severıty and CRP/Albumin Levels in Cases Wıth Covid-19 Pneumonia. J. Pioneer. Med. Sci..

[34] Taha S.I., Shata A.K., El-Sehsah E.M., Fouad S.H., Moussa A.H., Abdalgeleel S.A., Moustafa N.M., Youssef M.K. (2022). The Role of CRP, Interleukin-6 and Their Derived Immune-Inflammatory Indices in Early Prediction of Severity and Mortality of COVID-19 Patients. Microbes and Infectious Diseases.

[35] Torun A., Çakirca T.D., Çakirca G., Portakal R.D. (2021). The Value of C-Reactive Protein/Albumin, Fibrinogen/Albumin, and Neutrophil/Lymphocyte Ratios in Predicting the Severity of CoVID-19. Revista da Associação Médica Brasileira.

[36] Wang X., Xu Y., Huang H., Jiang D., Zhou C., Liao H., Chen X. (2020). An Increased Pretreatment C-Reactive Protein-to-Albumin Ratio Predicts Severe Novel Coronavirus-Infected Pneumonia. ResearchSquare.

[37] Wang H., Xing Y., Yao X., Li Y., Huang J., Tang J., Zhu S., Zhang Y., Xiao J. (2020). Retrospective Study of Clinical Features of COVID-19 in Inpatients and Their Association with Disease Severity. Med. Sci. Monit..

[38] Xing Y., Wang H., Yao X.H., Li Y., Huang J.T., Tang J., Zhu S., Liu Y.Q., Xiao J. (2020). Analysis of Factors for Disease Progression in 61 Patients with COVID-19 in Xiaogan, Hubei, China. Eur. Rev. Med. Pharmacol. Sci..

[39] Xue G., Gan X., Wu Z., Xie D., Xiong Y., Hua L., Zhou B., Zhou N., Xiang J., Li J. (2020). Novel Serological Biomarkers for Inflammation in Predicting Disease Severity in Patients with COVID-19. Int. Immunopharmacol..

[40] Yılmaz Demirci N., Uğraş Dikmen A., Taşçı C., Doğan D., Arslan Y., Öcal N., Taşar M., Bozlar U., Artuk C., Yılmaz G. (2021). Relationship between Chest Computed Tomography Findings and Clinical Conditions of Coronavirus Disease (COVID-19): A Multicentre Experience. Int. J. Clin. Pract..

[41] Li Y., Li H., Song C., Lu R., Zhao Y., Lin F., Han D., Chen L., Pan P., Dai M. (2021). Early Prediction of Disease Progression in Patients with Severe COVID-19 Using C-Reactive Protein to Albumin Ratio. Dis. Markers.

[42] Gozdas H.T., Kayis S.A., Damarsoy T., Ozsari E., Turkoglu M., Yildiz I., Demirhan A. (2022). Multi-Inflammatory Index as a Novel Mortality Predictor in Critically Ill COVID-19 Patients. J. Intensive Care Med..

[43] Yazar S., Arslan K., Sehit S., Varank İ., Baş S., Şehit S. (2022). The Relationship between the Prognostic Nutritional Index and the Clinical Course of COVID-19: A Single-Center Experience. J. Med. Palliat. Care.

[44] Hocanli I., Kabak M. (2022). The Clinical Importance of C-Reactive Protein to Albumin Ratio (CAR) in Patients Diagnosed with COVID-19. J. Contemp. Med..

[45] Prasad S., Patel S., Behera A.K., Gitismita N., Shah S., Nanda R., Mohapatra E. (2022). Early Biochemical Markers in Predicting the Clinical Outcome of COVID-19 Patients Admitted in Tertiary Care Hospital in Chhattisgarh, India. J. Lab. Physicians.

[46] Katkat F., Kalyoncuoglu M., Karahan S., Ozcan S., Atam Tasdemir Z., Kucuk H., Karabulut U., Guner A., Biter I., Nihan F. (2022). The Predictive Ability of the C-Reactive Protein to Albumin Ratio as A Mortality Predictor in Hospitalized Severe SARS-CoV-2 Infected Patients with Cardiovascular Diseases. Med. Bull. Haseki..

[47] Ergenc Z., Ergenç H., Araç S., Usanmaz M., Alkılınç E., Kaya G., Karacaer C., Nalbant A., Kaya T. (2022). Novel Biochemical Prognostic Indicators in COVID-19: Can CRP/Albumin, Urea/Albumin, and LDH/Albumin Ratios Be Used to Predict Mortality and Length of Hospitalization?. Med. Sci. Discov..

[48] Çalışkan Z., Bozdağ E., Sönmez S., Dağıstanlı S., Bulut N., Dinçer Y. (2022). Assessment of 7 Inflammatory Indexes as an Early Predictor of COVID-19 Severity. Cerrah-Med. J..

[49] Çelikkol A., Güzel E.Ç., Doğan M., Erdal B., Yilmaz A. (2022). C-Reactive Protein-to-Albumin Ratio as a Prognostic Inflammatory Marker in COVID-19. J. Lab. Physicians.

[50] Yazıcı M.M., Altuntaş G., Aygün A., Nalbant E. (2022). The Value of the C-Reactive Protein/Albumin and Fibrinogen/Albumin Ratios in Predicting Disease Severity and Mortality in Elderly COVID-19 Patients. Med. Sci. Discov..

[51] Choudhary S., Sharma K., Silakari O. (2021). The Interplay between Inflammatory Pathways and COVID-19: A Critical Review on Pathogenesis and Therapeutic Options. Microb. Pathog..

[52] Hariyanto T.I., Japar K.V., Kwenandar F., Damay V., Siregar J.I., Lugito N.P.H., Tjiang M.M., Kurniawan A. (2021). Inflammatory and Hematologic Markers as Predictors of Severe Outcomes in COVID-19 Infection: A Systematic Review and Meta-Analysis. Am. J. Emerg. Med..

[53] Ghahramani S., Tabrizi R., Lankarani K.B., Kashani S.M.A., Rezaei S., Zeidi N., Akbari M., Heydari S.T., Akbari H., Nowrouzi-Sohrabi P. (2020). Laboratory Features of Severe vs. Non-Severe COVID-19 Patients in Asian Populations: A Systematic Review and Meta-Analysis. Eur. J. Med. Res..

[54] Kilercik M., Demirelce Ö., Serdar M.A., Mikailova P., Serteser M. (2021). A New Haematocytometric Index: Predicting Severity and Mortality Risk Value in COVID-19 Patients. PLoS ONE.

[55] Zhou W., Zhang G.L. (2019). C-Reactive Protein to Albumin Ratio Predicts the Outcome in Renal Cell Carcinoma: A Meta-Analysis. PLoS ONE.

[56] Wang Y., Hu X., Huang Y., Xu W.Y., Wu Y.M., Li P.F., Che G.W. (2020). Prognostic Value of the C-Reactive Protein to Albumin Ratio in Esophageal Cancer: A Systematic Review and Meta-Analysis. Kaohsiung. J. Med. Sci..

[57] Zang Y., Fan Y., Gao Z. (2020). Pretreatment C-Reactive Protein/Albumin Ratio for Predicting Overall Survival in Pancreatic Cancer: A Meta-Analysis. Medicine.

[58] Fan Y., Xiang S., Dai Z., Zou C., Wang X., Gao Z. (2019). Prognostic Significance of C-Reactive Protein to Albumin Ratio in Colorectal Cancer Patients: A Meta-Analysis. Int. J. Colorectal Dis..

[59] Wong T.C., Su H.Y., Chen Y.T., Wu P.Y., Chen H.H., Chen T.H., Hsu Y.H., Yang S.H. (2016). Ratio of C-Reactive Protein to Albumin Predicts Muscle Mass in Adult Patients Undergoing Hemodialysis. PLoS ONE.

[60] Kaplan M., Duzenli T., Tanoglu A., Cakir Guney B., Onal Tastan Y., Bicer H.S. (2020). Presepsin:Albumin Ratio and C-Reactive Protein:Albumin Ratio as Novel Sepsis-Based Prognostic Scores: A Retrospective Study. Wien. Klin. Wochenschr..

[61] Oh T.K., Song I.A., Lee J.H. (2018). Clinical Usefulness of C-Reactive Protein to Albumin Ratio in Predicting 30-Day Mortality in Critically Ill Patients: A Retrospective Analysis. Sci. Rep..

[62] Bai M., Wu Y., Ji Z., Wang S., Lin Z., Pan S., Huang K. (2019). Prognostic Value of C-Reactive Protein/Albumin Ratio in Neurocritically Ill Patients. Minerva Anestesiol..

[63] Ita K. (2021). Coronavirus Disease (COVID-19): Current Status and Prospects for Drug and Vaccine Development. Arch. Med. Res..

[64] Parasher A. (2021). COVID-19: Current Understanding of Its Pathophysiology, Clinical Presentation and Treatment. Postgrad Med. J..

[65] Wang H., Chang Y., Cui Z.Z., Liu Z.J., Ma S.F., Ma S.F. (2020). Admission C-Reactive Protein-to-Albumin Ratio Predicts the 180-Day Mortality of AIDS-Related Pneumocystis Pneumonia. AIDS Res. Hum. Retroviruses.

[66] Duman H., Çinier G., Bakırcı E.M., Duman H., Şimşek Z., Hamur H., Değirmenci H., Emlek N. (2019). Relationship Between C-Reactive Protein to Albumin Ratio and Thrombus Burden in Patients with Acute Coronary Syndrome. Clin. Appl. Thromb. Hemost..

[67] Kocatürk M., Kocatürk Ö. (2019). Assessment of Relationship between C-Reactive Protein to Albumin Ratio and 90-Day Mortality in Patients with Acute Ischaemic Stroke. Neurol. Neurochir. Pol..

[68] Süleymanoğlu M., Burak C., Gümüşdağ A., Yesin M., Rencüzoğulları İ., Karabağ Y., Çağdaş M., Çap M. (2020). Assessment of the Relation between C-Reactive Protein to Albumin Ratio and the Severity and Complexity of Peripheral Arterial Disease. Vascular.

[69] Yayla K.G., Yayla Ç. (2021). C-Reactive Protein-to-Albumin Ratio and Progression of Abdominal Aortic Aneurysm. Angiology.

[70] Aksoy F., Uysal D., Ibrişim E. (2020). Relationship between C-Reactive Protein/Albumin Ratio and New-Onset Atrial Fibrillation after Coronary Artery Bypass Grafting. Revista da Associacao Medica Brasileira (1992).

[71] Babapoor-Farrokhran S., Gill D., Walker J., Rasekhi R.T., Bozorgnia B., Amanullah A. (2020). Myocardial Injury and COVID-19: Possible Mechanisms. Life Sci..

[72] Long B., Brady W.J., Koyfman A., Gottlieb M. (2020). Cardiovascular Complications in COVID-19. Am. J. Emerg. Med..

[73] Bridwell R., Long B., Gottlieb M. (2020). Neurologic Complications of COVID-19. Am. J. Emerg. Med..

[74] Nannoni S., de Groot R., Bell S., Markus H.S. (2021). Stroke in COVID-19: A Systematic Review and Meta-Analysis. Int. J. Stroke.

[75] Yılmaz P.Ö., Karacan E. (2021). The Effects of C-Reactive Protein/Albumin Ratio and Hematologic Parameters on Predicting the Prognosis for Emergency Surgical Patients in Intensive Care. Ulus Travma Acil. Cerrahi. Derg..

[76] Xu H., Hu L., Wei X., Niu J., Gao Y., He J., Hou J. (2019). The Predictive Value of Preoperative High-Sensitive C-Reactive Protein/Albumin Ratio in Systemic Inflammatory Response Syndrome After Percutaneous Nephrolithotomy. J. Endourol..

[77] D’Amico G., Garcia-Tsao G., Pagliaro L. (2006). Natural History and Prognostic Indicators of Survival in Cirrhosis: A Systematic Review of 118 Studies. J. Hepatol..

[78] Kodner C. (2009). Nephrotic Syndrome in Adults: Diagnosis and Management—PubMed. Am. Fam. Physician.

[79] Oikonomou T., Goulis I., Kiapidou S., Tagkou N., Akriviadis E., Papatheodoridis G., Cholongitas E. (2020). The Significance of C-Reactive Protein to Albumin Ratio in Patients with Decompensated Cirrhosis. Ann. Gastroenterol..

