# Supplementary material for: C-Reactive Protein-to-Albumin Ratio and Clinical Outcomes in COVID-19 Patients: A Systematic Review and Meta-Analysis
Source: Trop Med Infect Dis. 2022 Aug 16;7(8):186. doi: 10.3390/tropicalmed7080186 (PMC9414550; doi:10.3390/tropicalmed7080186)
Supplement: Supplementary file 1 [file tropicalmed-07-00186-s001.zip › tropicalmed-1831519-Supplementary.pdf]

Tropical Medicine and Infectious Disease

MDPI

Supplementary Materials: **C-Reactive protein-to-albumin ratio and clinical outcomes in COVID-19 patients: A systematic review and meta-analysis**

File S1: Search Strategy

PUBMED

Albumin (#1)

Albumin [MH] OR Albumins [MH] OR Serum Albumin [MH] OR Albumin, Serum [MH] OR Plasma Albumin [MH] OR Albumin [TIAB] OR Albumins [TIAB] OR “Serum Albumin” [TIAB] OR “Plasma Albumin” [TIAB] OR Albumin [OT] OR Albumins [OT] OR “Serum Albumin” [OT] OR “Plasma Albumin” [OT]

C-Reactive Protein (#2)

C-Reactive Protein [MH] OR C Reactive Protein [MH] OR hsCRP [MH] OR High Sensitivity C-Reactive Protein [MH] OR High Sensitivity C Reactive Protein [MH] OR hs-CRP [MH] OR “C-Reactive Protein” [TIAB] OR “C Reactive Protein” [TIAB] OR hsCRP [TIAB] OR “High Sensitivity C-Reactive Protein” [TIAB] OR “High Sensitivity C Reactive Protein” [TIAB] OR hs-CRP [TIAB] OR “C-Reactive Protein” [OT] OR “C Reactive Protein” [OT] OR hsCRP [OT] OR “High Sensitivity C-Reactive Protein” [OT] OR “High Sensitivity C Reactive Protein” [OT] OR hs-CRP [OT]

C-Reactive Protein to Albumin Ratio (#3)

“C-Reactive Protein/Albumin ratio” [OT] OR “C-Reactive Protein/Albumin index” [OT] OR “Ratio C-Reactive Protein/Albumin” [OT] OR “Index C-Reactive Protein/Albumin” [OT] OR “C-Reactive Protein to Albumin ratio” [OT] OR “C-Reactive Protein-to Albumin ratio” [OT] OR “C-Reactive Protein to-Albumin ratio” [OT] OR “C-Reactive Protein-to-Albumin ratio” [OT] OR “C-Reactive Protein to Albumin index” [OT] OR “C-Reactive Protein-to Albumin index” [OT] OR “C-Reactive Protein to-Albumin index” [OT] OR “C-Reactive Protein-to-Albumin index” [OT] OR “Ratio C-Reactive Protein to Albumin” [OT] OR “Ratio C-Reactive Protein-to Albumin” [OT] OR “Ratio C-Reactive Protein to-Albumin” [OT] OR “Ratio C-Reactive Protein-to-Albumin” [OT] OR “Index C-Reactive Protein to Albumin” [OT] OR “Index C-Reactive Protein-to Albumin” [OT] OR “Index C-Reactive Protein to-Albumin” [OT] OR “Index C-Reactive Protein-to-Albumin” [OT] OR "CRP/Albumin Ratio" [OT] OR "CRP/Albumin Index" [OT] OR "Ratio CRP/Albumin" [OT] OR "Index CRP/Albumin" [OT] OR "CRP to Albumin Ratio" [OT] OR "CRP-to Albumin Ratio" [OT] OR "CRP to-Albumin Ratio" [OT] OR "CRP-to-Albumin Ratio" [OT] OR "CRP-to-Albumin-Ratio" [OT] OR "Ratio CRP to Albumin" [OT] OR "Ratio CRP-to Albumin" [OT] OR "Ratio CRP to-Albumin" [OT] OR "Ratio CRP-to-Albumin" [OT] OR "Ratio-CRP-to-Albumin" [OT] OR "CRP to Albumin Index" [OT] OR "CRP-to Albumin Index" [OT] OR "CRP to-Albumin Index" [OT] OR "CRP-to-Albumin Index" [OT] OR "CRP-to-Albumin-Index" [OT] OR "Index CRP to Albumin" [OT] OR "Index CRP-to Albumin" [OT] OR "Index CRP to-Albumin" [OT] OR "Index CRP-to-Albumin" [OT] OR "Index-CRP-to-Albumin" [OT]

Covid-19 (#4)

COVID 19 [MH] OR COVID-19 Virus Disease [MH] OR COVID 19 Virus Disease [MH] OR COVID-19 Virus Diseases [MH] OR Disease, COVID-19 Virus [MH] OR Virus Disease, COVID-19 [MH] OR COVID-19 Virus Infection [MH] OR COVID 19 Virus Infection [MH] OR COVID-19 Virus Infections [MH] OR Infection, COVID-19 Virus [MH] OR

Virus Infection, COVID-19 [MH] OR 2019-nCoV Infection [MH] OR 2019 nCoV Infection [MH] OR 2019-nCoV Infections [MH] OR Infection, 2019-nCoV [MH] OR Coronavirus Disease-19 [MH] OR Coronavirus Disease 19 [MH] OR 2019 Novel Coronavirus Disease [MH] OR 2019 Novel Coronavirus Infection [MH] OR 2019-nCoV Disease [MH] OR 2019 nCoV Disease [MH] OR 2019-nCoV Diseases [MH] OR Disease, 2019-nCoV [MH] OR COVID19 [MH] OR Coronavirus Disease 2019 [MH] OR Disease 2019, Coronavirus [MH] OR SARS Coronavirus 2 Infection [MH] OR SARS-CoV-2 Infection [MH] OR Infection, SARS-CoV-2 [MH] OR SARS CoV 2 Infection [MH] OR SARS-CoV-2 Infections [MH] OR COVID-19 Pandemic [MH] OR COVID 19 Pandemic [MH] OR COVID-19 Pandemics [MH] OR Pandemic, COVID-19 [MH] OR "COVID 19" [TIAB] OR "COVID-19 Virus Disease" [TIAB] OR "COVID 19 Virus Disease" [TIAB] OR "COVID-19 Virus Diseases" [TIAB] OR "COVID-19 Virus Infection" [TIAB] OR "COVID 19 Virus Infection" [TIAB] OR "COVID-19 Virus Infections" [TIAB] OR "Infection, COVID-19 Virus" [TIAB] OR "2019-nCoV Infection" [TIAB] OR "2019 nCoV Infection" [TIAB] OR "2019-nCoV Infections" [TIAB] OR "Coronavirus Disease-19" [TIAB] OR "Coronavirus Disease 19" [TIAB] OR "2019 Novel Coronavirus Disease" [TIAB] OR "2019 Novel Coronavirus Infection" [TIAB] OR "2019-nCoV Disease" [TIAB] OR "2019 nCoV Disease" [TIAB] OR "2019-nCoV Diseases" [TIAB] OR "COVID19" [TIAB] OR "Coronavirus Disease 2019" [TIAB] OR "Disease 2019, Coronavirus" [TIAB] OR "SARS Coronavirus 2 Infection" [TIAB] OR "SARS-CoV-2 Infection" [TIAB] OR "Infection, SARS-CoV-2" [TIAB] OR "SARS-CoV-2 Infections" [TIAB] OR "COVID-19 Pandemic" [TIAB] OR "COVID 19 Pandemic" [TIAB] OR "COVID-19 Pandemics" [TIAB] OR "COVID 19" [OT] OR "COVID-19 Virus Disease" [OT] OR "COVID 19 Virus Disease" [OT] OR "COVID-19 Virus Diseases" [OT] OR "COVID-19 Virus Infection" [OT] OR "COVID 19 Virus Infection" [OT] OR "COVID-19 Virus Infections" [OT] OR "Infection, COVID-19 Virus" [OT] OR "2019-nCoV Infection" [OT] OR "2019 nCoV Infection" [OT] OR "2019-nCoV Infections" [OT] OR "Coronavirus Disease-19" [OT] OR "Coronavirus Disease 19" [OT] OR "2019 Novel Coronavirus Disease" [OT] OR "2019 Novel Coronavirus Infection" [OT] OR "2019-nCoV Disease" [OT] OR "2019 nCoV Disease" [OT] OR "2019-nCoV Diseases" [OT] OR "COVID19" [OT] OR "Coronavirus Disease 2019" [OT] OR "Disease 2019, Coronavirus" [OT] OR "SARS Coronavirus 2 Infection" [OT] OR "SARS-CoV-2 Infection" [OT] OR "Infection, SARS-CoV-2" [OT] OR "SARS-CoV-2 Infections" [OT] OR "COVID-19 Pandemic" [OT] OR "COVID 19 Pandemic" [OT] OR "COVID-19 Pandemics" [OT]

Search Formula

((#1 AND #2) OR #3) AND #4

SCOPUS

(ALL (albumin OR albumins OR serum AND albumin OR albumin, AND serum OR plasma AND albumin OR albumin OR albumins OR "Serum Albumin" OR "Plasma Albumin" OR albumin OR albumins OR "Serum Albumin" OR "Plasma Albumin") AND ALL (c-reactive AND protein OR c AND reactive AND protein OR hscrp OR high AND sensitivity AND c-reactive AND protein OR high AND sensitivity AND c AND reactive AND protein OR hs-crp OR "C-Reactive Protein" OR "C Reactive Protein" OR hscrp OR "High Sensitivity C-Reactive Protein" OR "High Sensitivity C Reactive Protein" OR hs-crp OR "C-Reactive Protein" OR "C Reactive Protein" OR hscrp OR "High Sensitivity C-Reactive Protein" OR "High Sensitivity C Reactive Protein" OR hs-crp) OR ALL ("C-Reactive Protein/Albumin ratio" OR "C-Reactive Protein/Albumin index" OR "Ratio C-Reactive Protein/Albumin" OR "Index C-Reactive Protein/Albumin" OR "C-Reactive Protein to Albumin ratio" OR "C-Reactive Protein-to Albumin ratio" OR "C-Reactive Protein to-Albumin ratio" OR "C-Reactive Protein-to-Albumin ratio" OR "C-Reactive Protein to Albumin index" OR "C-Reactive Protein-to Albumin index" OR "C-Reactive Protein to-Albumin index" OR "C-Reactive Protein-to-Albumin index" OR "Ratio C-Reactive Protein

to Albumin" OR "Ratio C-Reactive Protein-to Albumin" OR "Ratio C-Reactive Protein to-Albumin" OR "Ratio C-Reactive Protein-to-Albumin" OR "Index C-Reactive Protein to Albumin" OR "Index C-Reactive Protein-to Albumin" OR "Index C-Reactive Protein to-Albumin" OR "Index C-Reactive Protein-to-Albumin" OR "CRP/Albumin Ratio" OR "CRP/Albumin Index" OR "Ratio CRP/Albumin" OR "Index CRP/Albumin" OR "CRP to Albumin Ratio" OR "CRP-to Albumin Ratio" OR "CRP to-Albumin Ratio" OR "CRP-to-Albumin Ratio" OR "CRP-to-Albumin-Ratio" OR "Ratio CRP to Albumin" OR "Ratio CRP-to Albumin" OR "Ratio CRP to-Albumin" OR "Ratio CRP-to-Albumin" OR "Ratio-CRP-to-Albumin" OR "CRP to Albumin Index" OR "CRP-to Albumin Index" OR "CRP to-Albumin Index" OR "CRP-to-Albumin Index" OR "CRP-to-Albumin-Index" OR "Index CRP to Albumin" OR "Index CRP-to Albumin" OR "Index CRP to-Albumin" OR "Index CRP-to-Albumin" OR "Index-CRP-to-Albumin") AND TITLE-ABS-KEY ("COVID-19" OR "COVID 19" OR "COVID-19 Virus Disease" OR "COVID 19 Virus Disease" OR "COVID-19 Virus Diseases" OR "Disease, COVID-19 Virus" OR "Virus Disease, COVID-19" OR "COVID-19 Virus Infection" OR "COVID 19 Virus Infection" OR "COVID-19 Virus Infections" OR "Infection, COVID-19 Virus" OR "Virus Infection, COVID-19" OR "2019-nCoV Infection" OR "2019 nCoV Infection" OR "2019-nCoV Infections" OR "Infection, 2019-nCoV" OR "Coronavirus Disease-19" OR "Coronavirus Disease 19" OR "2019 Novel Coronavirus Disease" OR "2019 Novel Coronavirus Infection" OR "2019-nCoV Disease" OR "2019 nCoV Disease" OR "2019-nCoV Diseases" OR "Disease, 2019-nCoV" OR "COVID19" OR "Coronavirus Disease 2019" OR "Disease 2019, Coronavirus" OR "SARS Coronavirus 2 Infection" OR "SARS-CoV-2 Infection" OR "Infection, SARS-CoV-2" OR "SARS CoV 2 Infection" OR "SARS-CoV-2 Infections" OR "COVID-19 Pandemic" OR "COVID 19 Pandemic" OR "COVID-19 Pandemics" OR "Pandemic, COVID-19"))

LILACS

C-Reactive Protein [Palabras] and Albumin [Palabras] and Covid [Palabras]

WEB OF SCIENCE

Query #1

Albumin OR Albumins OR Serum Albumin OR Albumin, Serum OR Plasma Albumin OR Albumin OR Albumins OR “Serum Albumin” OR “Plasma Albumin” OR Albumin OR Albumins OR “Serum Albumin” OR “Plasma Albumin” (All Fields)

Query #2

ALL=(C-Reactive Protein OR C Reactive Protein OR hsCRP OR High Sensitivity C-Reactive Protein OR High Sensitivity C Reactive Protein OR hs-CRP OR “C-Reactive Protein” OR “C Reactive Protein” OR hsCRP OR “High Sensitivity C-Reactive Protein” OR “High Sensitivity C Reactive Protein” OR hs-CRP OR “C-Reactive Protein” OR “C Reactive Protein” OR hsCRP OR “High Sensitivity C-Reactive Protein” OR “High Sensitivity C Reactive Protein” OR hs-CRP)

Query #3

ALL=(“C-Reactive Protein/Albumin ratio” OR “C-Reactive Protein/Albumin index” OR “Ratio C-Reactive Protein/Albumin” OR “Index C-Reactive Protein/Albumin” OR “C-Reactive Protein to Albumin ratio” OR “C-Reactive Protein-to Albumin ratio” OR “C-Reactive Protein to-Albumin ratio” OR “C-Reactive Protein-to-Albumin ratio” OR “C-Reactive Protein to Albumin index” OR “C-Reactive Protein-to Albumin index” OR “C-Reactive Protein to-Albumin index” OR “C-Reactive Protein-to-Albumin index” OR “Ratio C-Reactive Protein to Albumin” OR “Ratio C-Reactive Protein-to Albumin” OR “Ratio C-Reactive Protein to-Albumin” OR “Ratio C-Reactive Protein-to-Albumin” OR “Index C-

Reactive Protein to Albumin" OR "Index C-Reactive Protein-to Albumin" OR "Index C-Reactive Protein to-Albumin" OR "Index C-Reactive Protein-to-Albumin" OR "CRP/Albumin Ratio" OR "CRP/Albumin Index" OR "Ratio CRP/Albumin" OR "Index CRP/Albumin" OR "CRP to Albumin Ratio" OR "CRP-to Albumin Ratio" OR "CRP to-Albumin Ratio" OR "CRP-to-Albumin Ratio" OR "CRP-to-Albumin-Ratio" OR "Ratio CRP to Albumin" OR "Ratio CRP-to Albumin" OR "Ratio CRP to-Albumin" OR "Ratio CRP-to-Albumin" OR "Ratio-CRP-to-Albumin" OR "CRP to Albumin Index" OR "CRP-to Albumin Index" OR "CRP to-Albumin Index" OR "CRP-to-Albumin Index" OR "CRP-to-Albumin-Index" OR "Index CRP to Albumin" OR "Index CRP-to Albumin" OR "Index CRP to-Albumin" OR "Index CRP-to-Albumin" OR "Index-CRP-to-Albumin").

Query #4

ALL=("COVID-19" OR "COVID 19" OR "COVID-19 Virus Disease" OR "COVID 19 Virus Disease" OR "COVID-19 Virus Diseases" OR "Disease, COVID-19 Virus" OR "Virus Disease, COVID-19" OR "COVID-19 Virus Infection" OR "COVID 19 Virus Infection" OR "COVID-19 Virus Infections" OR "Infection, COVID-19 Virus" OR "Virus Infection, COVID-19" OR "2019-nCoV Infection" OR "2019 nCoV Infection" OR "2019-nCoV Infections" OR "Infection, 2019-nCoV" OR "Coronavirus Disease-19" OR "Coronavirus Disease 19" OR "2019 Novel Coronavirus Disease" OR "2019 Novel Coronavirus Infection" OR "2019-nCoV Disease" OR "2019 nCoV Disease" OR "2019-nCoV Diseases" OR "Disease, 2019-nCoV" OR "COVID19" OR "Coronavirus Disease 2019" OR "Disease 2019, Coronavirus" OR "SARS Coronavirus 2 Infection" OR "SARS-CoV-2 Infection" OR "Infection, SARS-CoV-2" OR "SARS CoV 2 Infection" OR "SARS-CoV-2 Infections" OR "COVID-19 Pandemic" OR "COVID 19 Pandemic" OR "COVID-19 Pandemics" OR "Pandemic, COVID-19")

Search Formula

(((#1) AND #2) OR #3) AND #4

OVIDMEDLINE

1 ..nlpx "query=Albumin OR Albumins OR Serum Albumin OR Albumin, Serum OR Plasma Albumin OR Albumin OR Albumins OR "Serum Albumin" OR "Plasma Albumin" OR Albumin OR Albumins OR "Serum Albumin" OR "Plasma Albumin"","desiredResults=10000","minHitsDivisor=7","permitHyponyms=NO","lowestVocabularySearchLevel=none","phrasesBroken=NO","speedWanted=NoHypos","comment=Incluyendo términos relacionados","elimEnable=NO","constraintMinTerms=2"

2 limit 1 to full text

3 ..nlpx "query=C-Reactive Protein OR C Reactive Protein OR hsCRP OR High Sensitivity C-Reactive Protein OR High Sensitivity C Reactive Protein OR hs-CRP OR "C-Reactive Protein" OR "C Reactive Protein" OR hsCRP OR "High Sensitivity C-Reactive Protein" OR "High Sensitivity C Reactive Protein" OR hs-CRP OR "C-Reactive Protein" OR "C Reactive Protein" OR hsCRP OR "High Sensitivity C-Reactive Protein" OR "High Sensitivity C Reactive Protein" OR hs-CRP","desiredResults=10000","minHitsDivisor=7","permitHyponyms=NO","lowestVocabularySearchLevel=none","phrasesBroken=NO","speedWanted=NoHypos","comment=Incluyendo términos relacionados","elimEnable=NO","constraintMinTerms=2"

4 limit 3 to full text

5 ..nlpx "query="C-Reactive Protein/Albumin ratio" OR "C-Reactive Protein/Albumin index" OR "Ratio C-Reactive Protein/Albumin" OR "Index C-Reactive Protein/Albumin" OR "C-Reactive Protein to Albumin ratio" OR "C-Reactive Protein-to Albumin ratio" OR "C-Reactive Protein to-Albumin ratio" OR "C-Reactive Protein-to-Albumin ratio" OR "C-Reactive Protein to Albumin index" OR "C-Reactive Protein-to Albumin in-

dex” OR “C-Reactive Protein to-Albumin index” OR “C-Reactive Protein-to-Albumin index” OR “Ratio C-Reactive Protein to Albumin” OR “Ratio C-Reactive Protein-to Albumin” OR “Ratio C-Reactive Protein to-Albumin” OR “Ratio C-Reactive Protein-to-Albumin” OR “Index C-Reactive Protein to Albumin” OR “Index C-Reactive Protein-to Albumin” OR “Index C-Reactive Protein to-Albumin” OR “Index C-Reactive Protein-to-Albumin” OR "CRP/Albumin Ratio" OR "CRP/Albumin Index" OR "Ratio CRP/Albumin" OR "Index CRP/Albumin" OR "CRP to Albumin Ratio" OR "CRP-to Albumin Ratio" OR "CRP to-Albumin Ratio" OR "CRP-to-Albumin Ratio" OR "CRP-to-Albumin-Ratio" OR "Ratio CRP to Albumin" OR "Ratio CRP-to Albumin" OR "Ratio CRP to-Albumin" OR "Ratio CRP-to-Albumin" OR "Ratio-CRP-to-Albumin" OR "CRP to Albumin Index" OR "CRP-to Albumin Index" OR "CRP to-Albumin Index" OR "CRP-to-Albumin Index" OR "CRP-to-Albumin-Index" OR "Index CRP to Albumin" OR "Index CRP-to Albumin" OR "Index CRP to-Albumin" OR "Index CRP-to-Albumin" OR "Index-CRP-to-Albumin” ","desiredResults=10000","minHitsDivisor=7","permitHyponyms=NO","lowestVocabularySearchLevel=none","phrasesBroken=NO","speedWanted=NoHypos","comment=Incluyendo términos relacionados","elimEnable=NO","constraintMinTerms=2"

6 limit 5 to full text

7 ..nlpx "query=“COVID-19” OR “COVID 19” OR “COVID-19 Virus Disease” OR “COVID 19 Virus Disease” OR “COVID-19 Virus Diseases” OR “Disease, COVID-19 Virus” OR “Virus Disease, COVID-19” OR “COVID-19 Virus Infection” OR “COVID 19 Virus Infection” OR “COVID-19 Virus Infections” OR “Infection, COVID-19 Virus” OR “Virus Infection, COVID-19” OR “2019-nCoV Infection” OR “2019 nCoV Infection” OR “2019-nCoV Infections” OR “Infection, 2019-nCoV” OR “Coronavirus Disease-19” OR “Coronavirus Disease 19” OR “2019 Novel Coronavirus Disease” OR “2019 Novel Coronavirus Infection” OR “2019-nCoV Disease” OR “2019 nCoV Disease” OR “2019-nCoV Diseases” OR “Disease, 2019-nCoV” OR “COVID19” OR “Coronavirus Disease 2019” OR “Disease 2019, Coronavirus” OR “SARS Coronavirus 2 Infection” OR “SARS-CoV-2 Infection” OR “Infection, SARS-CoV-2” OR “SARS CoV 2 Infection” OR “SARS-CoV-2 Infections” OR “COVID-19 Pandemic” OR “COVID 19 Pandemic” OR “COVID-19 Pandemics” OR “Pandemic, COVID-19”","desiredResults=10000","minHitsDivisor=7","permitHyponyms=NO","lowestVocabularySearchLevel=none","phrasesBroken=NO","speedWanted=NoHypos","comment=Incluyendo términos relacionados","elimEnable=NO","constraintMinTerms=2"

8 limit 7 to abstracts

9 2 and 4

10 6 or 9

11 8 and 10

WHO’S GLOBAL RESEARCH ON CORONAVIRUS DISEASE (COVID-19) DATABASE

tw:(C-Reactive Protein/Albumin ratio) AND collection:("01-internacional" OR "04-international_org" OR "09-preprints")

EMBASE

Query *Results*

#5 #3 AND #4

#4 #1 OR #2

#3 'covid-19':ab,ti OR 'covid 19':ab,ti OR 'covid-19 virus disease':ab,ti OR 'covid 19 virus disease':ab,ti OR 'covid-19 virus diseases':ab,ti OR 'disease, covid-19 virus':ab,ti OR 'virus disease, covid-19':ab,ti OR 'covid-19 virus infection':ab,ti OR 'covid 19 virus infection':ab,ti OR 'covid-19 virus infections':ab,ti OR 'infection, covid-19 virus':ab,ti OR 'virus infection, covid-19':ab,ti OR '2019-ncov infection':ab,ti OR '2019 ncov infection':ab,ti OR '2019-ncov infections':ab,ti OR 'infection, 2019-ncov':ab,ti OR 'coronavirus disease-19':ab,ti

OR 'coronavirus disease 19':ab,ti OR '2019 novel coronavirus disease':ab,ti OR '2019 novel coronavirus infection':ab,ti OR '2019-ncov disease':ab,ti OR '2019 ncov disease':ab,ti OR '2019-ncov diseases':ab,ti OR 'disease, 2019-ncov':ab,ti OR 'covid19':ab,ti OR 'coronavirus disease 2019':ab,ti OR 'disease 2019, coronavirus':ab,ti OR 'sars coronavirus 2 infection':ab,ti OR 'sars-cov-2 infection':ab,ti OR 'infection, sars-cov-2':ab,ti OR 'sars cov 2 infection':ab,ti OR 'sars-cov-2 infections':ab,ti OR 'covid-19 pandemic':ab,ti OR 'covid 19 pandemic':ab,ti OR 'covid-19 pandemics':ab,ti OR 'pandemic, covid-19':ab,ti

#2 'C-Reactive Protein/Albumin ratio" OR "C-Reactive Protein/Albumin index" OR "Ratio C-Reactive Protein/Albumin" OR "Index C-Reactive Protein/Albumin" OR "C-Reactive Protein to Albumin ratio" OR "C-Reactive Protein-to Albumin ratio" OR "C-Reactive Protein to-Albumin ratio" OR "C-Reactive Protein-to-Albumin ratio" OR "C-Reactive Protein to Albumin index" OR "C-Reactive Protein-to Albumin index" OR "C-Reactive Protein to-Albumin index" OR "C-Reactive Protein-to-Albumin index" OR "Ratio C-Reactive Protein to Albumin" OR "Ratio C-Reactive Protein-to Albumin" OR "Ratio C-Reactive Protein to-Albumin" OR "Ratio C-Reactive Protein-to-Albumin" OR "Index C-Reactive Protein to Albumin" OR "Index C-Reactive Protein-to Albumin" OR "Index C-Reactive Protein to-Albumin" OR "Index C-Reactive Protein-to-Albumin" OR "CRP/Albumin Ratio" OR "CRP/Albumin Index" OR "Ratio CRP/Albumin" OR "Index CRP/Albumin" OR "CRP to Albumin Ratio" OR "CRP-to Albumin Ratio" OR "CRP to-Albumin Ratio" OR "CRP-to-Albumin Ratio" OR "CRP-to-Albumin-Ratio" OR "Ratio CRP to Albumin" OR "Ratio CRP-to Albumin" OR "Ratio CRP to-Albumin" OR "Ratio CRP-to-Albumin" OR "Ratio-CRP-to-Albumin" OR "CRP to Albumin Index" OR "CRP-to Albumin Index" OR "CRP to-Albumin Index" OR "CRP-to-Albumin Index" OR "CRP-to-Albumin-Index" OR "Index CRP to Albumin" OR "Index CRP-to Albumin" OR "Index CRP to-Albumin" OR "Index CRP-to-Albumin" OR "Index-CRP-to-Albumin" '

#1 ('albumin, serum':ab,ti OR albumin:ab,ti OR albumins:ab,ti OR 'serum albumin':ab,ti OR 'plasma albumin':ab,ti) AND ('globulins, serum':ab,ti OR globulin:ab,ti OR globulins:ab,ti OR 'serum globulins':ab,ti OR pseudoglobulins:ab,ti OR euglobulins:ab,ti)

Table S1. : PRISMA Checklist.

Section and Topic	Item #	Checklist item	Location where item is reported
**TITLE**			
Title	1	Identify the report as a systematic review.	Title Page
**ABSTRACT**			
Abstract	2	See the PRISMA 2020 for Abstracts checklist.	Title Page
**INTRODUCTION**			
Rationale	3	Describe the rationale for the review in the context of existing knowledge.	Line 23 and 29 of the introduction section
Objectives	4	Provide an explicit statement of the objective(s) or question(s) the review addresses.	Line 29 and 32 of the introduction section
**METHODS**			
Eligibility criteria	5	Specify the inclusion and exclusion criteria for the review and how studies were grouped for the syntheses.	Subheading 2.3
Information sources	6	Specify all databases, registers, websites, organisations, reference lists and other sources searched or consulted to identify studies. Specify the date when each source was last searched or consulted.	Subheading 2.2
Search strategy	7	Present the full search strategies for all databases, registers and websites, including any filters and limits used.	Subheading 2.3 and Supplementary Appendix 1
Selection process	8	Specify the methods used to decide whether a study met the inclusion criteria of the review, including how many reviewers screened each record and each report retrieved, whether they worked independently, and if applicable, details of automation tools used in the process.	Subheading 2.4

Section and Topic	Item #	Checklist item	Location where item is reported
Data collection process	9	Specify the methods used to collect data from reports, including how many reviewers collected data from each report, whether they worked independently, any processes for obtaining or confirming data from study investigators, and if applicable, details of automation tools used in the process.	Subheading 2.5
Data items	10a	List and define all outcomes for which data were sought. Specify whether all results that were compatible with each outcome domain in each study were sought (e.g. for all measures, time points, analyses), and if not, the methods used to decide which results to collect.	Subheading 2.7
	10b	List and define all other variables for which data were sought (e.g. participant and intervention characteristics, funding sources). Describe any assumptions made about any missing or unclear information.	Subheading 2.7
Study risk of bias assessment	11	Specify the methods used to assess risk of bias in the included studies, including details of the tool(s) used, how many reviewers assessed each study and whether they worked independently, and if applicable, details of automation tools used in the process.	Subheadings 2.6 and 2.8
Effect measures	12	Specify for each outcome the effect measure(s) (e.g. risk ratio, mean difference) used in the synthesis or presentation of results.	Subheading 2.7
Synthesis methods	13a	Describe the processes used to decide which studies were eligible for each synthesis (e.g. tabulating the study intervention characteristics and comparing against the planned groups for each synthesis (item #5)).	Subheading 2.7
	13b	Describe any methods required to prepare the data for presentation or synthesis, such as handling of missing summary statistics, or data conversions.	Subheading 2.7
	13c	Describe any methods used to tabulate or visually display results of individual studies and syntheses.	Subheading 2.7
	13d	Describe any methods used to synthesize results and provide a rationale for the choice(s). If meta-analysis was performed, describe the model(s), method(s) to identify the presence and extent of statistical heterogeneity, and software package(s) used.	Subheading 2.7
	13e	Describe any methods used to explore possible causes of heterogeneity among study results (e.g. subgroup analysis, meta-regression).	Subheading 2.7
	13f	Describe any sensitivity analyses conducted to assess robustness of the synthesized results.	Subheading 2.7

Section and Topic	Item #	Checklist item	Location where item is reported
Reporting bias assessment	14	Describe any methods used to assess risk of bias due to missing results in a synthesis (arising from reporting biases).	Subheading 2.7
Certainty assessment	15	Describe any methods used to assess certainty (or confidence) in the body of evidence for an outcome.	Subheading 2.7
**RESULTS**			
Study selection	16a	Describe the results of the search and selection process, from the number of records identified in the search to the number of studies included in the review, ideally using a flow diagram.	Subheading 3.1
	16b	Cite studies that might appear to meet the inclusion criteria, but which were excluded, and explain why they were excluded.	Subheading 3.1
Study characteristics	17	Cite each included study and present its characteristics.	Subheading 3.2
Risk of bias in studies	18	Present assessments of risk of bias for each included study.	Subheadings 3.3 and 3.6
Results of individual studies	19	For all outcomes, present, for each study: (a) summary statistics for each group (where appropriate) and (b) an effect estimate and its precision (e.g. confidence/credible interval), ideally using structured tables or plots.	Subheadings 3.3 and 3.5
Results of syntheses	20a	For each synthesis, briefly summarise the characteristics and risk of bias among contributing studies.	Subheadings 3.3 and 3.5
	20b	Present results of all statistical syntheses conducted. If meta-analysis was done, present for each the summary estimate and its precision (e.g. confidence/credible interval) and measures of statistical heterogeneity. If comparing groups, describe the direction of the effect.	Subheadings 3.3 and 3.5
	20c	Present results of all investigations of possible causes of heterogeneity among study results.	Subheadings 3.3 and 3.5
	20d	Present results of all sensitivity analyses conducted to assess the robustness of the synthesized results.	Subheadings 3.3 and 3.5

Section and Topic	Item #	Checklist item	Location where item is reported
Reporting biases	21	Present assessments of risk of bias due to missing results (arising from reporting biases) for each synthesis assessed.	Subheadings 3.3 and 3.5
Certainty of evidence	22	Present assessments of certainty (or confidence) in the body of evidence for each outcome assessed.	Subheadings 3.3 and 3.5
**DISCUSSION**			
Discussion	23a	Provide a general interpretation of the results in the context of other evidence.	Line 1-3 of the Discussion section
	23b	Discuss any limitations of the evidence included in the review.	Paragraph 6 of the Discussion section
	23c	Discuss any limitations of the review processes used.	Paragraph 6 of the Discussion section
	23d	Discuss implications of the results for practice, policy, and future research.	Paragraph 6 of the Discussion section
**OTHER INFORMATION**			
Registration and protocol	24a	Provide registration information for the review, including register name and registration number, or state that the review was not registered.	Subheading 2.1
	24b	Indicate where the review protocol can be accessed, or state that a protocol was not prepared.	Subheading 2.1
	24c	Describe and explain any amendments to information provided at registration or in the protocol.	Subheading 2.1
Support	25	Describe sources of financial or non-financial support for the review, and the role of the funders or sponsors in the review.	Page25 in Statements and Declarations
Competing interests	26	Declare any competing interests of review authors.	Page25 in Statements and Declarations

Section and Topic	Item #	Checklist item	Location where item is reported
Availability of data, code and other materials	27	Report which of the following are publicly available and where they can be found: template data collection forms; data extracted from included studies; data used for all analyses; analytic code; any other materials used in the review.	Page25 in Statements and Declarations

Table S2. : Newcastle - Ottawa Quality assessment scale for included studies.

NEWCASTLE - OTTAWA QUALITY ASSESSMENT SCALE FOR COHORT STUDIES										
STUDY	**SELECTION**				**COMPARABILITY**	**OUTCOME**				
	**Representativeness of the exposed cohort**	**Selection of the non-exposed cohort**	**Ascertainment of exposure**	**Demonstration that outcome of interest was not present at start of study**	**Comparability of Cohorts on the Basis of the Design or Analysis Maximum : ☆☆**	**Assessment of outcome**	**Was follow-up long enough for outcomes to occur**	**Adequacy of follow up of cohorts**	**SCORE**	**Evidence quality**
Zhang M et .al	☆	☆	☆		☆	☆		☆	*6*	Low Risk of bias
Karakoyun I et.al		☆	☆	☆		☆	☆	☆	*6*	Low Risk of bias
El-Shabrawy M et.al	☆	☆	☆			☆	☆	☆	*6*	Low Risk of bias
Wang X et.al		☆	☆			☆	☆	☆	*5*	Moderate risk of bias
Xue G et .al		☆	☆			☆	☆	☆	*5*	Moderate risk of bias
Wang H et.al		☆	☆			☆		☆	*4*	Moderate risk of bias
Torun A et.al	☆	☆	☆			☆	☆	☆	*6*	Low Risk of bias
Gemcioglu E et.al		☆	☆	☆		☆	☆	☆	*6*	Low Risk of bias
Yılmaz N et.al		☆	☆			☆	☆	☆	*5*	Moderate risk of bias

Şirikçi V et.al		☆	☆			☆	☆		*4*	Moderate risk of bias
Xing Y et.al	☆	☆	☆	☆		☆	☆	☆	*7*	Low Risk of bias
Alisik M et.al		☆	☆		☆	☆	☆	☆	*6*	Low Risk of bias
Taha S et.al		☆	☆			☆		☆	*4*	Moderate risk of bias
Az A et .al		☆	☆		☆	☆	☆	☆	*6*	Low Risk of bias
Açıksarı G et.al	☆	☆	☆			☆	☆	☆	*6*	Low Risk of bias
Saylik F et.al		☆	☆	☆		☆			*4*	Moderate risk of bias
Kalabin A et.al		☆	☆	☆	☆		☆		*5*	Moderate risk of bias
Paliogiannis P et.al		☆	☆		☆	☆	☆	☆	*6*	Low Risk of bias
Kalyon S et . al	☆	☆	☆			☆	☆	☆	*6*	Low Risk of bias
Ozdemir IH et.al				☆	☆	☆		☆	*4*	Moderate risk of bias
Ozdemir S et.al			☆	☆	☆	☆	☆	☆	*6*	Low Risk of bias
Acehan S et.al			☆	☆	☆	☆	☆		*5*	Moderate risk of bias
Li Yi et.al		☆	☆			☆	☆	☆	*5*	Moderate risk of bias
Çelikkol A et.al		☆	☆			☆	☆	☆	*5*	High Risk of Bias
Çalışkan Z et.al	☆		☆			☆	☆	☆	*5*	High Risk of Bias
Ergenç Z et.al		☆	☆			☆	☆	☆	*5*	High Risk of Bias
Yazıcı et.al		☆	☆			☆	☆	☆	*5*	High Risk of Bias
Katkat F et.al		☆	☆			☆	☆	☆	*5*	High Risk of Bias
Prasad S et.al		☆	☆			☆	☆	☆	*5*	High Risk of Bias
Hocanlı I et.al	☆		☆			☆	☆	☆	*5*	High Risk of Bias

Arslan K et.al		☆	☆				☆	☆	☆	*5*	High Risk of Bias
Gozdas H et.al		☆	☆				☆	☆	☆	*5*	High Risk of Bias

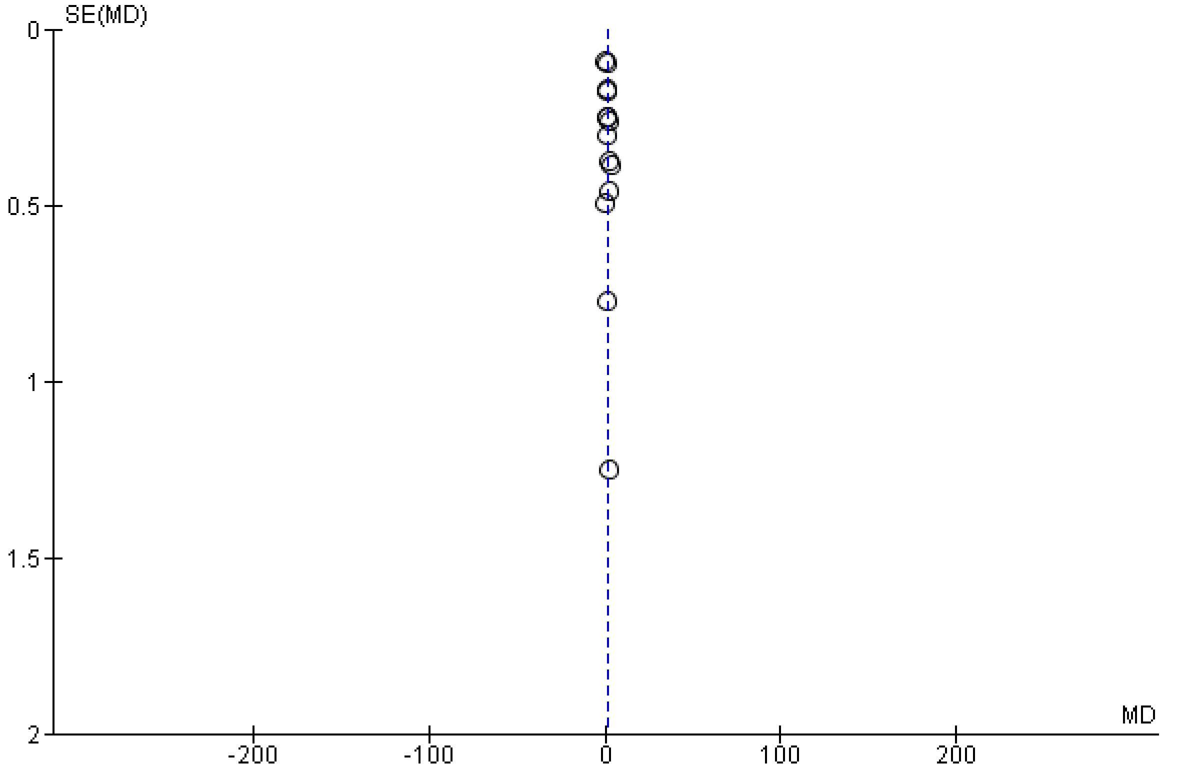

Figure S1. Funnel Plot of the studies that evaluated CAR values in severe vs. non-severe COVID-19 patients.

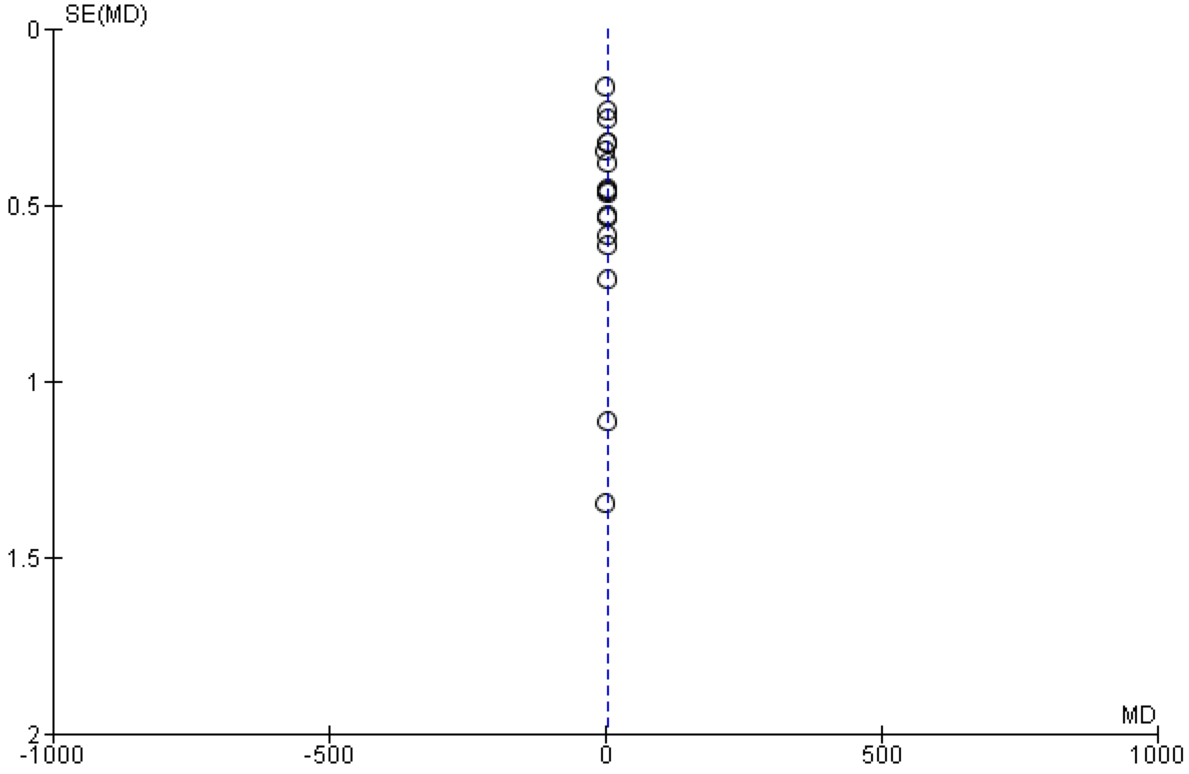

Figure S2. : Funnel Plot of the studies that evaluated CAR values in non-survivors vs. survivors COVID-19 patients.
